# A Functional Role of Fibroblast Growth Factor Receptor 1 (FGFR1) in the Suppression of Influenza A Virus Replication

**DOI:** 10.1371/journal.pone.0124651

**Published:** 2015-04-24

**Authors:** Xin Liu, Chengcai Lai, Keyu Wang, Li Xing, Penghui Yang, Qing Duan, Xiliang Wang

**Affiliations:** 1 State Key Laboratory of Pathogen and Biosecurity, Beijing Institute of Microbiology and Epidemiology, Beijing, China; 2 302 Military Hospital, Beijing, China; University of Georgia, UNITED STATES

## Abstract

Influenza A virus causes annual epidemics and occasional pandemics in humans. Here, we investigated four members of the fibroblast growth factor receptor (FGFR) family; FGFR1 to 4, and examined their expression patterns in human lung epithelial cells A549 with influenza A virus infection. We identified a functional role of FGFR1 in influenza A/Puerto Rico/8/1934 (PR8) and A/Anhui/01/2005 (H5N1) virus replication. Our results showed that FGFR1 silencing by siRNA interference promoted influenza A/PR8 and H5N1 virus replication in A549 cells, while lentivirus-mediated exogenous FGFR1 expression significantly suppressed influenza A virus replication; however, FGFR4 did not have the same effects. Moreover, FGFR1 phosphorylation levels were downregulated in A549 cells by influenza A virus infection, while the repression of FGFR1 kinase using PD173074, a potent and selective FGFR1 inhibitor, could enhance virus replication. Furthermore, we found that FGFR1 inhibits influenza virus internalization, but not binding, during viral entry. These results suggested that FGFR1 specifically antagonizes influenza A virus replication, probably by blocking viral entry.

## Introduction

Influenza A virus causes epidemics of respiratory diseases in humans, leading to thousands of deaths annually. This subtype of pathogen has been thoroughly studied, and many aspects including viral entry, and replication, as well as host cell-virus interactions are well-characterized. During the influenza virus life cycle, entry is the first essential process by which viral genomes are delivered from extracellular virions into the cell nucleus for viral replication [1, 2], which is an ideal target to block infection. Influenza virus enters the cell by receptor-mediated endocytosis after binding to the sialic acid receptor [3–5]. There is increasing evidence that viral attachment to the cell surface by influenza hemagglutinin (HA) is not sufficient for endocytotic routes [6, 7]. The adaptor protein Epsin-1 is recruited for clathrin-mediated viral entry [8]. Specific cellular signaling by receptor tyrosine kinases, including epidermal growth factor receptor (EGFR) and c-Met receptor, is required. siRNA-induced EGFR knockdown and the inhibition of kinase activity by small molecule inhibitors leads to impaired influenza virus uptake into cells [9]. In turn, host cells express antiviral factors to defend against influenza virus entry [10]. For example, the interferon-inducible transmembrane (IFITM) proteins 1 to 3 restrict an entry step of influenza A viral replication [11, 12].

The existence of these cellular factors suggests that other receptors play a role in viral entry. Fibroblast growth factor receptor (FGFR) 1 to 4, known as the tyrosine kinase receptor superfamily, regulates many biological processes including differentiation, proliferation, development, and angiogenesis [13–15]. FGFR1 is a co-receptor during adeno-associated virus 2 (AAV) infection for successful viral entry into the host cell [16]. FGFR4 knockdown reduces influenza A/WSN pseudotyped particle entry, and an FGFR4 inhibitor attenuates WSN virus replication [10]. These results increase our understanding of the role of FGFR family members in the life cycle of viral infection. Differential expression and structural complexity of the FGFR family may generate functional diversity in response to different ligands, which is crucial for regulating normal physiological processes *in vivo* [17]. However, it remains unclear how the expression of FGFR 1 to 4 are regulated by influenza A virus infection, and whether FGFR 1 to 4 have the same effect on influenza A (H1N1 or H5N1) virus replication requires further study.

In this study, we investigated the differential expression of four FGFR family members in A549 cells with PR8 infection and the functional roles of FGFR1 and FGFR4 on PR8 or H5N1 virus replication. We found that RNAi-induced FGFR1 gene silencing (not FGFR4) significantly elevated PR8 and H5N1 virus replication, while lentivirus-mediated FGFR1 overexpression reduced virus replication, which suggested that FGFR1 specifically suppressed influenza A virus replication. In addition, inhibition of FGFR1 kinase activity could enhance influenza A virus replication. During the early stages of infection, FGFR1 could inhibit viral internalization steps, which may account for the suppressive effect of FGFR1 on virus replication.

## Materials and Methods

### Viruses and cells

Two influenza A (H1N1) virus strains: A/Puerto Rico/8/1934 (PR8) and A/Anhui/01/2005 (H5N1) were used in this study. The strains were propagated in 9- to 11-day-old specific-pathogen-free (SPF) chicken embryos. Virus titers were determined based on the 50% tissue infectious dose (TCID_50_) assay using Madin-Darby canine kidney (MDCK) cells purchased from the ATCC according to the Reed-Muench method [18]. The human lung adenocarcinoma epithelial cell line A549 from the ATCC was cultured in Dulbecco’s Modified Eagle’s medium (DMEM) supplemented with 10% (v/v) fetal bovine serum (FBS) and 100 units penicillin-streptomycin ml^-1^ at 37°C in a humidified atmosphere of 5% (v/v) CO_2_. All experiments with live H5N1 virus were conducted in approved bio-safety level 3 (BSL-3) laboratory facilities.

### RNA isolation and real-time quantitative PCR

Total RNA was extracted from cultured cells with TRIzol reagent [19]. A total of 2 μg of RNA was treated with RNase-free DNase I (Promega) for 30 min at 37°C. Complementary DNA (cDNA) was generated by reverse transcription with 8 μl of 5× PrimeScript RT Master Mix (TaKaRa) for 15 min at 37°C. A total of 10 μl of SYBR Premix Ex Taq II (TaKaRa) were mixed with cDNA and specific primers to a total volume of 20 μl. The primer pairs *FGFR1*, *FGFR2*, *FGFR3*, *FGFR4*, influenza virus *M1*, and *GAPDH* (Table 1) were designed using Primer Premier software 5.0 (Premier Biosoft International, Palo Alto, CA, USA) and synthesized by Invitrogen. Real-time PCR was performed in triplicate wells of a 96-well reaction plate on an ABI 7300 Real-Time PCR System (Applied Biosystems). The quantification data were analyzed with ABI 7300 SDS software v.1.3.

**Table 1:** Primer pairs used for real-time PCR.

Gene	Forward	Reverse
***FGFR1***	5′- CGCCCCTGTACCTGGAGATCATCA -3′	5′- TTGGTACCACTCTTCATCTT -3′;
***FGFR2***	5′- GCCTGGAAGAGAAAAGGAGATTAC -3′	5′- GGATGACTGTTACCACCATACA -3′;
***FGFR3***	5′- CATCCGGCAGACGTACACGC -3′	5′- ACTGTACACCTTGCAGTGGA -3′;
***FGFR4***	5′- GTGCCCTCGGACCGCGGCACATAC -3′	5′- TCCGAAGCTGCTGCCGTTGATG -3′;
***M1***	5′- AAGACCAATCCTGTCACCTCTG -3′	5′- CAAAACGTCTACGCTGCAGTCC -3′
***GAPDH***	5′- GGTGGTCTCCTCTGACTTCAACA -3′	5′- GTTGCTGTAGCCAAATTCGTTGT -3′

Primer pairs of human *FGFR1*, *FGFR2*, *FGFR3*, *FGFR4*, influenza virus *M1*, and *GAPDH* were designed using Primer 5.0 and presented in Table 1.

### Western blotting assay

Protein extracts from cell lysates were obtained with RIPA buffer mixed protease inhibitor cocktail (Thermo scientific). Protein extractions were boiled in 4× Protein SDS-PAGE Loading Buffer (Takara) for 5 min and resolved on a SDS-PAGE, and then transferred onto 0.45-μm NC membrane (GE Whatman). Membranes with total FGFR proteins were blocked by 5% (w/v) non-fat dry milk (GE Healthcare), while those with phosphorylated FGFR1 protein were treated with 5% (w/v) bovine serum albumin (BSA) for 1 h at room temperature and then incubated overnight at 4°C with primary antibodies including anti-FGFR1 polyclonal antibody (1:500 dilution, Cell Signaling Technology), anti-phosphorylated FGFR1 polyclonal antibody (1:1000 dilution, Lianke Biotechnology Ltd.), anti-FGFR4 monoclonal antibody (1:1000 dilution, Abcam; Epitomics), and anti-β-actin monoclonal antibody (1:5000 dilution, Sigma-Aldrich). After three washes with TBS containing 0.1% (v/v) Triton X-100 (TBST), they were incubated with goat anti-rabbit or anti-mouse horseradish peroxidase (HRP)-conjugated secondary antibody (1:5000 dilution, Cell Signaling Technology) for 1 h at room temperature. Final detection of protein was performed using the Signal Boost Immunoreaction Enhancer Kit (Merck Millipore). Protein levels were quantified using Quantity One software (Bio-Rad).

### FGFR knockdown with small interfering RNAs (siRNAs)

All siRNA transfection was performed as described previously [20]. SiRNAs against two different regions of each target genes were synthesized by RiboBio (Guangzhou, China). For *FGFR1* and *FGFR4* silencing, A549 cells were transfected with 20 nM siRNA using Lipofectamine RNAiMax (Invitrogen) according to the manufacturer's instructions. The medium was replaced with fresh complete medium 6 h after transfection, and the cells were harvested 48 h after transfection for FGFR repression efficiency detection by real-time PCR and Western blotting.

### Indirect immunofluorescence microscopy

After 48 h of siRNA transfection, A549 cells were infected with PR8 virus. Four hours later, A549 cells were fixed with 4% (w/v) paraformaldehyde at room temperature for 15–20 min, as described previously [21]. After two washes with PBS, cells were permeabilized with PBS containing 10% (v/v) FBS, 3% (w/v) BSA, and 0.5% (v/v) Triton X-100 for 15 min, after which they were incubated with an anti-influenza A virus NP primary antibody (1:1000 dilution, Merck Millipore) overnight at 4°C. After two washes with PBS containing 0.2% (w/v) BSA and 0.1% (v/v) Triton X-100, cells were incubated with Alexa Fluor 488-labeled secondary antibodies (Invitrogen) at room temperature for 1 h. Cell nuclei were stained with Hoechst 33342 (Sigma) for 10 min followed by three PBS washes. Images were captured using a Nikon Eclipse TE2000-U inverted microscope. Analysis was performed with Image Pro Plus software (Media Cybernetics). Number of total cells and virus-infected positive cells in each of three areas (× 250 field) was counted, and then the average count of the three fields was recorded.

### Lentivirus-mediated FGFR overexpression in A549 cells

DNA fragments corresponding to the coding sequence of human *FGFR1* and *FGFR4* genes were obtained from A549 cDNA by PCR amplification and subcloned into plasmid pWPXL between restriction sites *Pac*I and *Nde*I or *Eco*RI. The primer sequences used were as follows: *FGFR1* forward, 5’- CGGTTAATTAACCATGTGGAGCTGGAAGTGCC -3’ and reverse, 5’- CTGCATATGTCAGCGGCGTTTGAGTCC -3’; *FGFR4* forward, 5’- GCTTAATTAACCATGCGGCTGCTGCTGGCCCTGT -3’ and reverse, 5’- TTGGAATTCCTGTCTGCACCCCAGACCCGAAG -3’. The 293T cells were transfected with pWPXL-R1 or pWPXL-R4 and two other helper plasmids. After 48 h transfection, recombinant lentivirus was obtained from 293T cells and filtered through 0.45-μm filters. Empty lentivirus vector was used as a control. For lentivirus infection, A549 cells were incubated with diluted virus supernatant (>95% infection efficiency) supplemented with 6 μg polybrene (Sigma-Aldrich) ml^-1^ for 8 h. Medium was then replaced with fresh complete medium and cultured for a further 40 h. Cells were harvested for FGFR ectopic expression efficiency determination by real-time PCR and Western blotting assay.

### Virus binding and internalization assay

For influenza virus binding, A549 cells with FGFR1, FGFR4, or GFP overexpression were pretreated with or without 0.01 units of sialidase (Sigma-Aldrich) ml^-1^ at 37°C for 8 h [9], and then pre-cooled for 10 min, incubated with PR8 or H5N1 virus at a multiplicity of infection (MOI) of 1 at 4°C for 1 h, and finally washed twice with ice-cold PBS [1]. Cell lysates were prepared at 4°C with ice-cold RIPA buffer containing protease inhibitor cocktail. For virus internalization, cells with prebound virus were warmed to 37°C for 30 min and then washed with acidic cold PBS-HCl (pH 1.3) for detection of internalized virus particles, but not attached particles [9]. Cell lysates were obtained to determine influenza virus NP levels by Western blotting.

### Statistical analyses

Statistical analysis of the results was performed in GraphPad Prism software 4.0 (GraphPad Software, CA). Data was shown as means ± s.e.m. Statistical significance was assessed by one-way analysis of variance (ANOVA). Statistical significances are indicated as * *P*<0.05; ** *P*<0.01; *** *P*<0.001.

## Results

### Expression patterns of FGFR family members in A549 cells with influenza A (H1N1) virus infection

**Fig 1:**
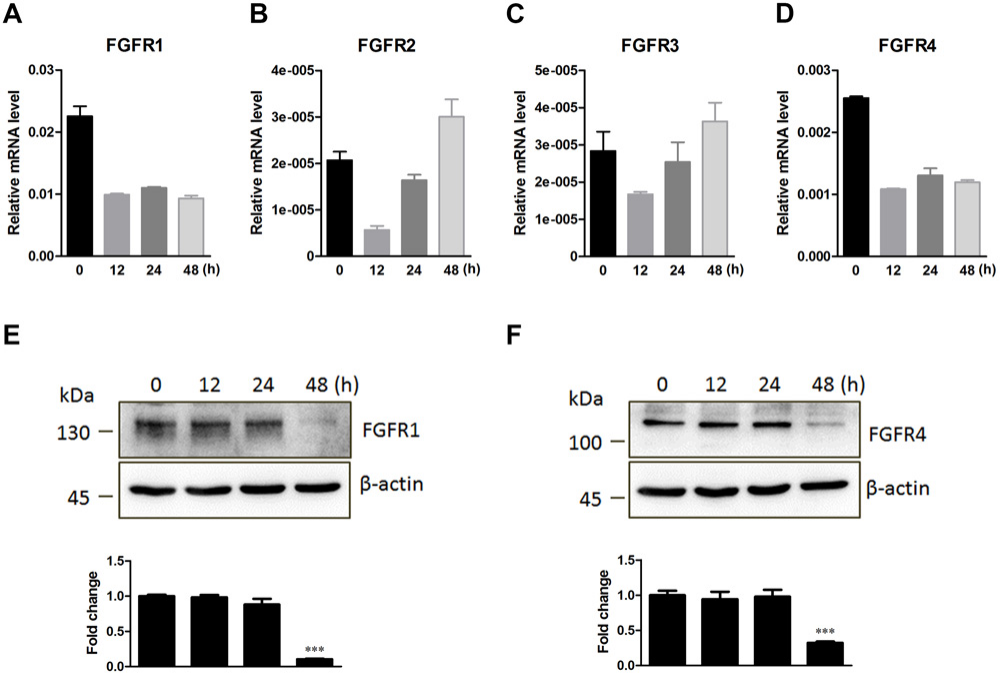
Time-course analysis of FGFR family member expression in A549 cells infected with influenza A/PR/8/34 virus. (**A**-**D**) A549 cells were infected with PR8 virus at an MOI of 1 for the indicated times. FGFR mRNA levels were detected using real-time PCR analysis. The mRNA expression of FGFR1 (**A**), FGFR2 (**B**), FGFR3 (**C**), and FGFR4 (**D**) relative to the reference gene GAPDH was calculated. (**E**, **F**) The lysates of A549 cells were obtained at the indicated times post-PR8 infection. Protein levels of FGFR1 (**E**) and FGFR4 (**F**) were determined by Western blotting using specific antibodies. Densitometric analysis relative to β-actin levels was expressed as fold change. All graphs present the means ± s.e.m. (n = 3). Values of P<0.001 *** were considered statistically highly significant.

We investigated four members of the FGFR family (FGFR1, FGFR2, FGFR3, and FGFR4) based on mRNA expression in human lung epithelial cells A549 at the indicated time points during influenza A/PR8/8/34 virus infection (Fig 1A–1D). The results revealed the differential expression levels of four FGFR members in A549 cells: FGFR1 mRNA expression was highest, while FGFR4 was intermediate and FGFR2 and FGFR3 were lowest. The results showed that the FGFR1 and FGFR4 mRNA expression decreased sharply at 12 hpi, and then stabilized. PR8 virus infection also decreased FGFR2 and FGFR3 mRNA expression at 12 hpi, which increased subsequently. We next detected the protein levels of four FGFR members by Western blotting using specific antibodies. The results showed that the FGFR1 and FGFR4 protein levels in A549 cells significantly decreased at 48 hpi (Fig 1E and 1F), followed by mRNA expression levels (Fig 1A and 1D). However, FGFR2 and FGFR3 protein expression could hardly be detected within the sensitivity of our experiment, which was most likely due to low levels of mRNA expression in A549 cells.

### The expression repression of FGFR1 by siRNA interference increased influenza A virus replication

**Fig 2:**
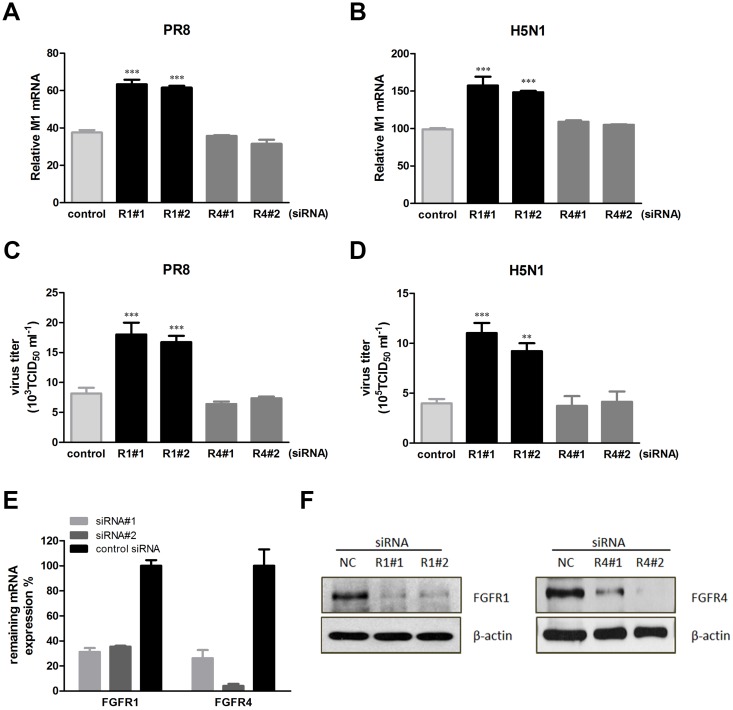
FGFR1 silencing by RNAi increased influenza A/PR8 and H5N1 virus replication. (**A**-**D**) A549 cells were transiently transfected with specific siRNA targeting FGFR1, FGFR4, or negative control siRNA. Forty-eight hours later, A549 cells were infected with PR8 virus at an MOI of 1. The cell culture supernatants and cell lysates were obtained at 24 hpi. Influenza virus M1 mRNA expression in A549 cells with PR8 (**A**) or H5N1 (**B**) infection was detected using real-time PCR. Progeny virus titers of PR8 (**C**) or H5N1 (**D**) were determined using MDCK cells with the TCID_50_ assay. (**E**) The knockdown efficiencies of FGFR1 and FGFR4 by target siRNA were tested using real-time PCR. (**F**) Protein expressions of FGFR1 and FGFR4 were detected using specific antibodies by Western blotting assay. All graphs represent the means ± s.e.m. (n = 3). Values of P<0.01 **and P<0.001 *** were considered statistically highly significant.

According to expression patterns of FGFR family members in A549 cells, we investigated the effect of FGFR1 and FGFR4 on influenza A/PR8 and H5N1 virus infection. A549 cells were transiently transfected with siRNAs for specific genetic interference, and then infected with PR8 or H5N1 virus. The mRNA levels of influenza virus M1 were detected using real-time PCR. The results showed that the M1 mRNA levels in PR8-infected A549 cells were markedly elevated by FGFR1 siRNA#1 and siRNA#2, but not by FGFR4 siRNA#1, siRNA#2, or control siRNA (Fig 2A). In addition, we determined progeny virus titers using MDCK cells with the TCID_50_ assay. Consistent with virus M1 mRNA expression, the viral titers were also increased in FGFR1 silenced cells (Fig 2C). Similar results were observed in H5N1-infected A549 cells (Fig 2B and 2D). The mRNA expression levels of FGFR1 and FGFR4 were analyzed using real-time PCR after 48 h transfection, and showed up to 60% knockdown by RNAi (Fig 2E). The effective repression of two proteins was also determined by Western blotting (Fig 2F). These results suggested that FGFR1 silencing promoted PR8 and H5N1 virus replication in A549 cells, while FGFR4 had no effect.

### FGFR1 silencing resulted in a significant elevation of influenza A virus infection efficiency

**Fig 3:**
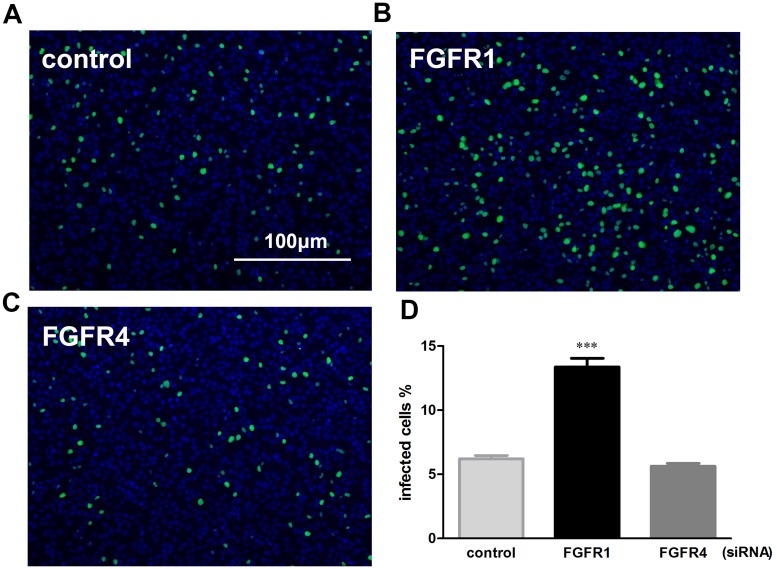
Specific siRNA target FGFR1 markedly increased PR8 virus entry at an early stage of the viral life cycle. (**A**-**C**) A549 cells were transfected with FGFR1 siRNA#1, FGFR4 siRNA#1, and negative control siRNA. After 48 h of transduction, A549 cells were incubated with PR8 virus at an MOI of 0.01 for 4 h, followed by indirect immunofluorescence assays. A549 cells were stained with anti-influenza A virus NP antibodies (green) and Hoechst 33342 (nucleus, blue). (**D**) The data of PR8-infected cells were presented as the percentages of NP-positive cells to the total number of cells. The bars represent the means ± s.e.m. (n = 3). P<0.001 *** were considered statistically highly significant.

Incoming vRNPs, containing nucleoprotein (NP)-encapsidated viral genomic RNAs with associated viral polymerase proteins (PA, PB1, and PB2), are mostly located in the nuclei at 4 hpi; after 7 hours, newly synthesized vRNPs are predominantly in the cytoplasm [22]. To investigate the effect of siRNA-mediated FGFR1 silencing on early stage of influenza virus infection, incoming vRNPs were detected at 4 h after PR8 virus infection using indirect immunofluorescence assays with anti-influenza A virus NP antibodies (Fig 3A–3C) [22, 23]. The results showed that the incoming vRNPs were mainly located in the nuclei of A549 cells at 4 hpi, while they could hardly be detected in the cytoplasm. The percentage of NP-positive cells to the total number of cells was then calculated (Fig 3D). Cells exposed to FGFR1 siRNA interference showed the highest percentage of NP-positive cells compared with FGFR4-repressed cells or the negative control. These results indicated that FGFR1 silencing significantly increased PR8 entry efficiency at an early stage of the viral life cycle.

### Influenza A virus replication could be reduced by lentivirus-mediated FGFR1 overexpression

**Fig 4:**
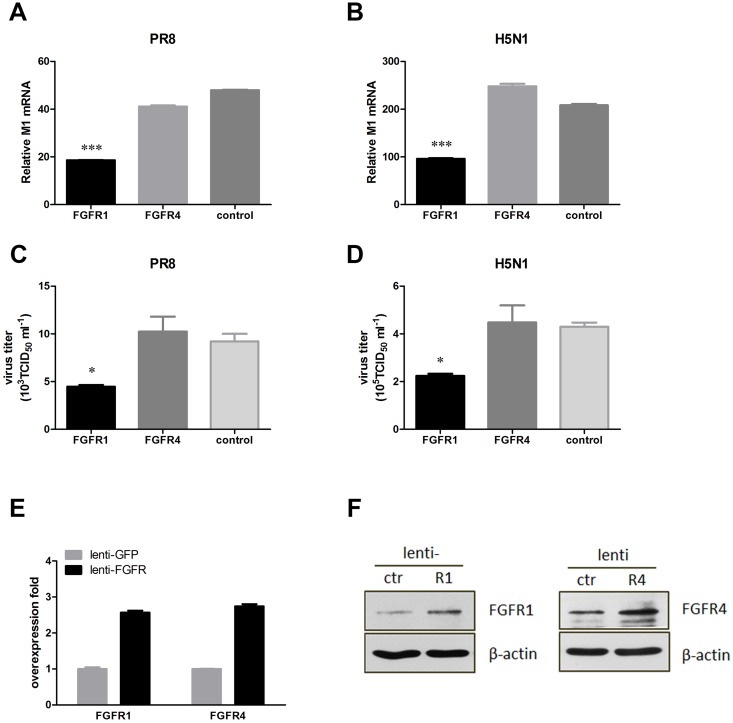
Lentivirus-mediated FGFR1 overexpression in A549 cells significantly decreased influenza A virus replication. A549 cells were infected with recombinant lentivirus expressing FGFR1, FGFR4, or GFP (as a control). After 48 h, A549 cells were infected with PR8 virus (MOI = 1). The supernatants and lysates of A549 cells were harvested after 24 h virus infection. (**A**, **B**) Influenza M1 mRNA levels in A549 cells with PR8 and H5N1 were detected using real-time PCR. (**C**, **D**) Progeny virus titers of PR8 and H5N1 were determined as described previously. (**E**, **F**) FGFR1 and FGFR4 expression efficiencies were detected using real-time PCR and Western blotting. All graphs present the means ± s.e.m. (n = 3). Values of P<0.05 * were considered statistically significant and P<0.001 *** statistically highly significant.

To confirm the effect of FGFR1 on influenza A virus infection, we explored whether overexpression of FGFR1 in A549 cells downregulated influenza A virus replication. Thus, recombinant lentivirus vectors for efficient delivery of exogenous FGFR1, FGFR4, or GFP to A549 cells were constructed. A549 cells with lentivirus-mediated FGFR1 or FGFR4 overexpression were infected with PR8 or H5N1 virus. The virus M1 mRNA levels and viral titers were then detected as described previously. As expected, FGFR1 significantly decreased virus M1 mRNA levels in PR8-infected A549 cells, but not FGFR4 or GFP (Fig 4A). Viral titers were also reduced in FGFR1-overexpressing cells, but not in the other two groups (Fig 4C). Similar results were observed in H5N1-infected A549 cells (Fig 4B and 4D). There was more than a 2.5-fold increase in mRNA expression of FGFR1 or FGFR4 in A549 cells (Fig 4E). The effective exogenous expression of FGFR1 or FGFR4 was also confirmed by Western blotting (Fig 4F). These results indicated that FGFR1 as a cellular antiviral factor could inhibit influenza A virus infection; meanwhile, influenza A virus may evade the inhibitory effect by downregulating FGFR1 protein levels.

### Treatment of PD173074, a FGFR kinase activity inhibitor, enhanced influenza A/PR8 virus replication

**Fig 5:**
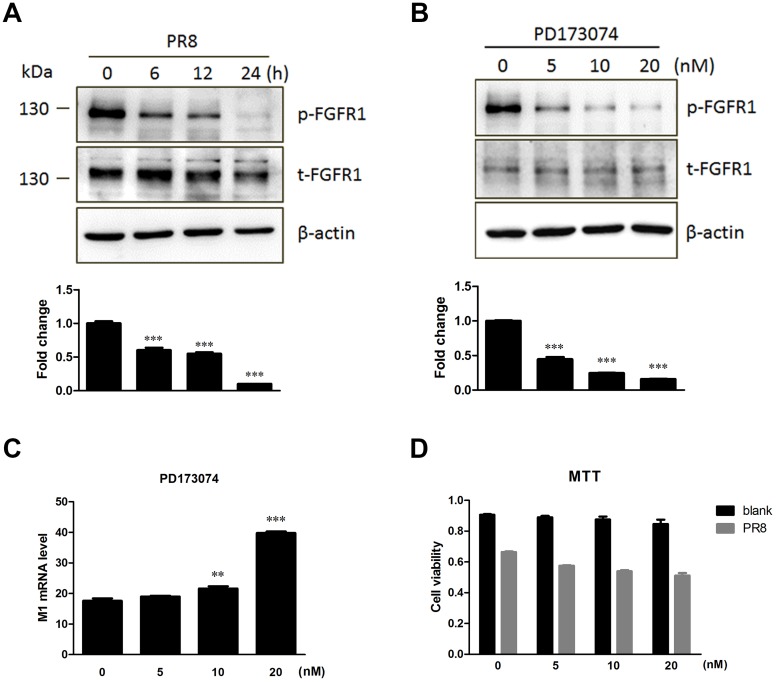
Repression of FGFR1 phosphorylation by the PD173074 inhibitor enhanced influenza A/PR8virus replication. (A) The lysates of A549 cells with PR8 virus infection were harvested for the indicated times. FGFR1 phosphorylation levels were determined by Western blotting with phospho-specific antibodies. Densitometric analysis of the phosphorylated/total FGFR1 ratio was shown as fold change. (B-D) A549 cells were pretreated with PD173074 (FGFR1 kinase inhibitor) at indicated concentrations for 30 min, and then infected with PR8 (indicated-dose inhibitor was also added to virus diluent). After 1 h, the medium was replaced with fresh medium supplemented with PD173074. (B) The cell lysates were collected at 12 hpi and then subjected to Western blotting for FGFR1 phosphorylation detection. The phosphorylated/total FGFR1 ratio indicated the fold change of FGFR1 phosphorylation. (C) Influenza M1 mRNA in PR8 infected-A549 cells with PD173074 treatment as indicated was detected by real-time PCR. (D) MTT assay of A549 cells with PD173074 treatment as indicated with or without PR8 infection. The results were expressed as means ± s.e.m. (n = 3). Values of P<0.05 * were considered statistically significant and P<0.001 *** considered statistically highly significant.

FGFR1 is a receptor tyrosine kinase. Tyrosine autophosphorylation of FGFR1 can be induced by extracellular stimuli [24]. Our previous results suggested that influenza A (H1N1) virus infection downregulated FGFR1 expression (Fig 1A and 1E), but whether influenza virus infection stimulated FGFR1 phosphorylation remains unknown. FGFR1 could inhibit influenza virus replication, but whether FGFR1 kinase activation had an effect on virus replication has not yet been explored. Next, the effect of influenza infection in A549 cells on FGFR1 phosphorylation for the indicated times was investigated (Fig 5A). The results showed that FGFR1 phosphorylation was obviously reduced by PR8 infection over time, which suggested that influenza A (H1N1) virus infection downregulated FGFR1 phosphorylation in A549 cells. To address the second question, PD173074 as a potent and highly selective inhibitor of the FGFR family that can inhibit autophosphorylation of FGFR1 in a dose-dependent manner with an IC50 in the range 1–5 nM [25] was used to verify the effect of FGFR1 kinase activity on virus replication. A549 cells were pretreated with PD173074 and then infected with PR8 virus. The results showed that FGFR1 phosphorylation in PR8-infected A549 cells was significantly suppressed by PD173074 in a dose-dependent manner (Fig 5B). There was little difference in M1 expression in A549 cells with 5 nM of PD173074 treatment; however, when the dose of PD173074 increased to 10 and 20 nM, virus M1 expression in A549 cells remarkably increased (Fig 5C). For cytotoxicity analysis, the viability of A549 cells with varying amounts of PD173074 with or without PR8 infection was measured using the MTT assay. The results showed that the range 5–20 nM of PD173074 did not induce significant cytotoxicity (Fig 5D). These results suggested that the repression of FGFR1 phosphorylation in A549 cells with PD173074 treatment could enhance influenza virus replication.

### FGFR1 overexpression inhibited influenza virus internalization, but not binding, during viral entry

**Fig 6:**
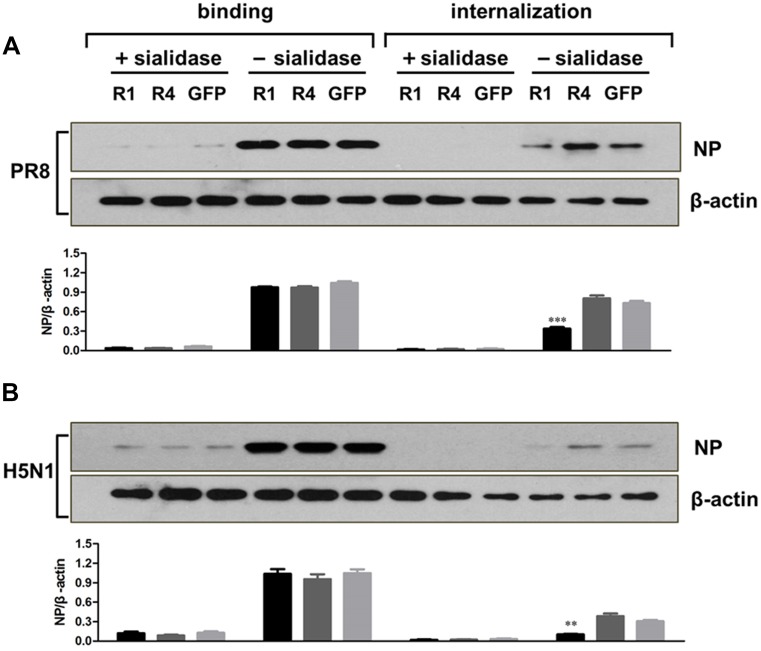
Lentivirus-delivered FGFR1 overexpression affected influenza A virus internalization, but not binding, during virus entry. A549 cells with FGFR1, FGFR4, or GFP overexpression were pretreated with or without sialidase, and then infected with influenza A/PR8 (**A**) or H5N1 (**B**). The procedures were described in detail in the Methods, virus binding and internalization assay. Influenza virus NP was detected by Western blotting using anti-influenza NP antibodies. β-actin protein was used as an internal control. The ratio of NP/β-actin was determined based on densitometric analysis. The results were expressed as means ± s.e.m. (n = 3). Values of P<0.05 * were considered statistically significant and P<0.01 ** highly significant.

Our previous results indicated that FGFR1 silencing promoted influenza virus entry at an early stage of the viral life cycle (Fig 3). We speculated that FGFR1 might play a suppressive role in virus binding and/or internalization. To further verify the potential effect of FGFR1, virus binding and internalization assays were performed. A549 cells with FGFR1, FGFR4, or GFP overexpression were pretreated with or without sialidase to prevent virus attachment and internalization, and then incubated with PR8 or H5N1 virus. During the entry process, virus bound to the cell surface at 4°C but was not internalized; after warming the cells to 37°C, virus began to internalize. The ratio of NP/β-actin was quantified and represented the amount of bound or internalized virus particles (Fig 6A and 6B). The results showed that the application of sialidase potently inhibited PR8 and H5N1 virus attachment and internalization. There was little difference in virus binding among three groups of cells with or without sialidase. However, in sialidase-untreated cells with FGFR1-overexpression, virus internalization was significantly reduced compared to the two other groups. The results suggested that FGFR1 specifically inhibited influenza A virus internalization, but not attachment to cells.

## Discussion

In this study, we found that FGFR1 silencing by RNAi remarkably increased IAV replication, while lentivirus-mediated FGFR1 overexpression effectively reduced IAV replication. Differences caused by FGFR1 silencing or overexpression were almost two-fold, which indicated that FGFR1 played a suppressive role in influenza A virus (IAV) PR8 and H5N1 replication. It is known that influenza virus attaches to the cell surface by binding of the HA protein, and then enters the cell through receptor-mediated endocytosis. Several studies have shown that influenza virus HA binding to sialic acid receptor is not sufficient to initiate virus entry. Host factors including dynamin, Epsin-1, EGFR, and PLC-γ1 etc. are known to be involved in this process and facilitate viral internalization [8, 9, 26, 27]. Our results suggested that FGFR1 knockdown notably elevated virus entry efficiency during the early stages of IAV infection; furthermore, FGFR1 could inhibit virus internalization, which indicated that host cells may have evolved a specific strategy of utilizing plasma membrane as a barrier to block influenza virus entry.

Four FGFR members have distinct spatial patterns of distribution in normal human adult tissues [28]. Our results showed that abundant FGFR1 and FGFR4 mRNA transcripts and protein expressions were observed in A549 cells, while FGFR2 and FGFR3 with lower mRNA levels could not be detected at the protein level, which was indicative of the differential roles of FGFR family members in A549 cells. A previous study using genome-wide siRNA screening assays showed that FGFR4 knockdown reduced influenza A/WSN pseudotyped particle entry in A549 cells, and also that the FGFR4 inhibitor attenuated WSN virus replication [10]. In our study, FGFR4 knockdown had no significant effect on inhibiting PR8 and H5N1 virus replication; in contrast, we demonstrated that FGFR1 not FGFR4 remarkably suppressed PR8 and H5N1 virus replication. Since FGFR2 and FGFR3 were not further investigated in our study because of their undetectable protein expression in A549 cells, it remains possible that they play a role in influenza virus replication.

FGFR signaling pathways play critical roles in many aspects of biological processes. In this study, we found that both FGFR1 protein expression and kinase phosphorylation levels were downregulated by influenza A/PR8 infection, which may be related to pathogenesis of influenza virus-induced disease. FGFR contains a highly conserved region between the first and second IgG-like domains, which exhibit extreme sequence similarity to the amino termini of the HA1 chains of influenza A virus HA [29]. Deletion of this HA-containing region completely abolished FGFR function of responding to basic FGF (FGFR ligand), which suggested that the HA-containing region plays a crucial role in regulating ligand binding or receptor oligomerization. In addition, FGFR dimerization is required for receptor tyrosine kinase activation [24, 30]; thus, an interaction may exist between influenza virions and the HA-containing region of FGFR1, by which receptor dimerization was blocked and kinase activity was inhibited.

Receptor tyrosine kinase signaling is known to play important roles in influenza A virus (IAV) replication [22]. The inhibition of tyrosine kinase activity with broad-range tyrosine kinase inhibitor genistein results in impaired IAV uptake into cells; EGFR kinase activation is required for efficient viral internalization. Besides, two other inhibitors, AG879 (known to inhibit the nerve growth factor receptor and epidermal growth factor receptor) and tyrphostin A9 (a selective inhibitor of the platelet-derived growth factor receptor) block multiple steps of IAV replication [9]. In our study, the inhibition of FGFR1 phosphorylation with a selective inhibitor PD173074 enhanced IAV replication.

Our results indicated that FGFR1 suppressed influenza A virus replication, probably by inhibiting viral internalization, but it remains unclear whether FGFR1 was implicated in other steps of the IAV life cycle. The mechanism of FGFR1-induced inhibition of virus internalization has not been fully explored. In this study, a functional role of FGFR1 on PR8 and H5N1 infection was found in A549 cells, but whether FGFR1 has a universal effect in other human cell lines requires further study.
